# ZCWPW1 is associated with late-onset Alzheimer's disease in Han Chinese: a replication study and meta-analyses

**DOI:** 10.18632/oncotarget.7945

**Published:** 2016-03-06

**Authors:** Yu Gao, Meng-Shan Tan, Hui-Fu Wang, Wei Zhang, Zi-Xuan Wang, Teng Jiang, Jin-Tai Yu, Lan Tan

**Affiliations:** ^1^ Department of Neurology, Qingdao Municipal Hospital, School of Medicine, Qingdao University, Qingdao, PR China; ^2^ Department of Emergency, Qingdao Municipal Hospital, School of Medicine, Qingdao University, Qingdao, PR China; ^3^ Department of Neurology, Nanjing First Hospital, Nanjing Medical University, Nanjing, PR China

**Keywords:** ZCWPW1, rs1476679, polymorphism, Alzheimer's disease

## Abstract

Recently, a large genome-wide association study (GWAS) has identified a novel variant (rs1476679) within *ZCWPW1* showing strong association with late-onset Alzheimer's disease (LOAD) in Caucasian. However, the effect of rs1476679 on other populations remains unclear. In order to explore whether rs1476679 is also associated with the LOAD risk in other ethnic groups, we recruited 2350 unrelated Northern Han Chinese subjects, which include 992 LOAD patients and 1358 healthy controls. Analysis of data from these subjects suggests that the rs1476679 polymorphism is significantly associated with the LOAD (genotype *P* = 0.017, allele *P* = 0.044). The logistic regression reveals the C allele at rs1476679 is a protective factor for LOAD in the dominant model (OR = 0.779, 95%CI = 0.659–0.921, *Pc* = 0.009) adjusting for gender, age and *APOE ε4* status. Furthermore, rs1476679 can decrease the AD risk (Dominant: OR = 0.733, 95%CI = 0.607–0.884, *Pc* = 0.006; Additive: OR = 0.820, 95%CI = 0.708–0.950, *Pc* = 0.048) in *APOE ε4* non-carriers after stratification. Furthermore, meta-analysis of 82525 individuals confirmed that rs1476679 within *ZCWPW1* decreased the risk of LOAD (OR = 0.91, 95%CI = 0.89–0.94). To summarize, the rs1476679 polymorphism in *ZCWPW1* is associated with LOAD in Northern Han Chinese population.

## INTRODUCTION

Alzheimer's disease (AD) is a genetically complex multifactorial neurodegenerative disorder and can be regarded as the most common type of dementia, defined by extensive neuronal and synapses loss, the formation of extracellular *amyloid-β* (*Aβ*) plaques as well as the accumulation of intracellular neurofibrillary tangles (NFTs) in the brain [1]. Even though AD influences 13% of the population older than 65 years and 43% older than 85 years, its underlying pathogenesis still remains unclear [2]. Previous studies identified that multiple rare mutations in the *APP, PSEN1, and PSEN2* genes contributes to early-onset familial AD. However, only the ε4 allele of the *apolipoprotein E* (*APOE*) gene increases the risk of late-onset AD (LOAD). Unfortunately, *APOE* only contributes to approximately 20% of LOAD risk, suggesting that numerous additional AD risk loci have not been identified so far. Previous GWAS identified some other genomic regions associated with LOAD, including *CLU, PICALM, CR1, BIN1, MS4A, CD2AP, CD33, EPHA1, ABCA7, TREM2,* etc. [3–10].

Recently, a large-scale GWAS study of AD, which included 74,046 individuals, adopted a two stage design and carried out a meta-analysis. 11 new discovered loci were found in this meta-analysis, *ZCWPW1* (rs1476679) can be seen as a member of them and have functional relevance [11]. He et al. applied solution NMR spectroscopy and determination of the 3D structure of *ZCWPW1* (encoding zinc finger, CW type with PWWP domain 1). *ZCWPW1* is a histone modification reader and is involved in epigenetic regulation [12]. *ZCWPW1* serves as an eQTL (expression quantitative trait loci) for *GATS*, *PILRB* and *TRIM4* and show indications for affecting binding of RFX3 and CTCF [13]. Besides, in a large LD block, candidate gene called *NYAP1* in the *ZCWPW1* region may activate the PI3K signaling pathway in neurons and thus inhibit LOAD incidence [11, 14]. *ZNF3* lies at the same locus on chromosome7 as *ZCWPW1*, interacts with BAG3 which is response for ubiquitin/proteasomal functions in protein degradation and binding of BACH1 whose target genes have roles in the oxidative stress response and control of the cell cycle [15]. Studies summarized above suggest that there has a specifically association between NFTs and *ZCWPW1* in LOAD.

However, note that this GWAS study was conduct only in Caucasian populations [11], and the effects of rs1476679 on other populations are yet unknown. Since variants and their frequencies of *ZCWPW1* in various ethnic groups might be different, replication is necessary to confirm the potential effects of *ZCWPW1* on other groups. In this study, we firstly conducted the genetic association study on rs1476679 within *ZCWPW1* in Han Chinese.

## RESULTS

We analyzed data from 2350 ethnic Northern Han Chinese subjects, which include 992 subjects (42%) with probable LOAD and 1358 healthy control subjects (58%) matched for age (age at onset for patients with LOAD compared with age at examination for control subjects; *P* = 0.067) and gender (*P* = 0.189). The demographic and clinical characteristics of AD and control subjects are summarized in Table 1. As expected, the scores of the Mini-Mental State Examination had significantly lower values for patients with LOAD (11.94 ± 6.21) than those for the control subjects (28.49 ± 1.09; *P* < 0.001). The possession of at least one *APOE ε4* allele was confirmed to increase the risk of developing LOAD (OR = 2.451, 95%CI = 1.995–3.011, *P* < 0.001). Then we examined the underlying association of the rs1476679 polymorphism with the LOAD susceptibility.

**Table 1:** The characteristics of the study population

	AD (*n* = 992)	Control (*n* = 1358)	*P* value	OR (95%CI)
Age at examination, years; mean ± SD	79.83 ± 6.69	75.49 ± 6.48	0.189*	
Age at onset, years; mean ± SD	75.17 ± 6.08			
Gender, *n* (%)			0.067	
Male	408 (41.1)	610 (44.9)		
Female	584 (58.9)	748 (55.1)		
MMSE score, mean ± SD	11.94 ± 6.21	28.49 ± 1.09	< 0.001	
*APOE ε4* status, *n* (%)			< 0.001	
*APOE ε4* (+)	284 (28.6)	191 (14.1)		2.451 (1.995–3.011)
*APOE ε4* (−)	708 (71.4)	1167 (85.9)		

Abbreviation: AD, Alzheimer's disease; Control, healthy controls; OR, odds ratio; CI, confidence interval; MMSE, Mini-Mental State Examination; *APOE*, apolipoprotein E; SD, standard deviation.

* *P* value was calculated with the age of onset for late-onset AD and age at examination for Control. Differences in the characteristics of age and MMSE score between the two groups were examined using Student's *t* test. Differences in gender and *APOE* ε4 frequency between AD patients and Control were assessed using the Pearson χ2 test.

Genotype distributions of the rs1476679 in *ZCWPW1* gene in controls exhibited the Hardy–Weinberg equilibrium (*P* > 0.05). Based on the minor allele (C) frequency in controls, our sample size had more than 95% power to detect the OR of 0.78 for LOAD between carriers and no-carriers, at a significance level (alpha) of 0.05. The allele and genotype distributions of rs1476679 AD patients and control subjects are shown in Table 2. The frequency of the minor allele C was lower in LOAD compared to the controls (28.9% vs. 31.7%). And there is significant difference between the LOAD patients and controls (OR= 0.879, 95%CI = 0.774–0.977, *P* = 0.044). Similarly, the genotypes of the LOAD patients were significantly different from those of the controls (*P* = 0.017). As showed in Table 3, rs1476679 strongly decreased the risk of LOAD in multivariate analysis under a dominant model (OR = 0.779, 95%CI = 0.659–0.921, *P* = 0.003, *P*c = 0.009) in total sample (adjusting for gender, age and *APOE ε4* status).

**Table 2:** Distribution of the rs1476679 alleles and genotypes in the AD cases and the controls

	N	Genotypes *n* (%)				Alleles *n* (%)		
		CC	CT	TT	P	C	T	P
*rs1476679*								
AD	992	82 (8.3)	410 (41.3)	500 (50.4)	0.017	574 (28.9)	1410 (71.1)	0.044
Controls	1358	110 (8.1)	640 (47.1)	608 (44.8)		860 (31.7)	1856 (68.3)	
*APOE ε4* (+)								
AD	284	20 (7.0)	134 (47.2)	130 (45.8)	0.049	174 (30.6)	394 (69.4)	0.385
Controls	191	4 (2.1)	99 (51.8)	88 (46.1)		107 (28.0)	275 (72.0)	
*APOE ε4* (−)								
AD	708	62 (8.8)	276 (39.0)	370 (52.3)	0.004	400 (28.3)	1016 (71.75)	0.010
Controls	1167	106 (9.1)	541 (46.4)	520 (44.6)		753 (32.3)	1581 (67.7)	

**Table 3:** Logistic regression analysis of rs1476679 polymorphisms

SNP	Total sample^a^					*APOE ε4* (+)^*b*^			*APOE ε4* (−)^b^		
	OR (95%CI)	*P*	*Pc*	*P*for APOE interaction	*Pc*	OR (95%CI)	*P*	*Pc*	OR (95%CI)	*P*	*Pc*
rs1476679											
Dominant	0.779 (0.659–0.921)	0.003*	0.009*	0.143		1.003 (0.692–1.453)	0.987		0.733 (0.607–0.884)	0.001*	0.006*
Additive	0.871 (0.763–0.995)	0.041*	0.123	0.055		1.159 (0.845–1.592)	0.360		0.820 (0.708–0.950)	0.008*	0.048*
Recessive	1.098 (0.811–1.486)	0.547		0.024*	0.072	3.531 (1.185–10.519)	0.023*	0.138	0.951 (0.684–1.321)	0.764	

Abbreviation: *Pc*, the corrected *P* value multiplied by 3 (the number of genetic models) in the total sample and by 6 in the *APOE* ε4 allele carrier and noncarrier subgroups as a Bonferroni adjustment.

*Pc* was only calculated when the *P* value was less than 0.05.

a Ajusted for age, gender, and *APOE ε4* statues.

b Ajusted for age and gender.

* *P* < 0.05, significant values.

Furthermore, to test the influence of the interaction between rs1476679 and *APOE ε4* genotype on the risk of developing LOAD, we analyzed them in logistic regression models. No interaction between rs1476679 and *APOE ε4* status was observed after Bonferroni adjustment. To further investigate whether the presence of the *APOE ε4* allele significantly modified the association of rs1476679 with LOAD, these data were stratified based on the presence/absence of the *APOE ε4*. The genotype and allele distributions in rs1476679 between LOAD patients and controls are significantly different (genotype *P* = 0.004, allele *P* = 0.010) from subjects without *APOE ε4* allele. In the subjects with *APOE ε4* allele, the genotypic distributions (*P* = 0.049) have differences between LOAD and controls. However, no significant differences were observed in the allelic distributions (*P* = 0.385) between these two groups (Table 2). Multivariate analysis also reveals that significant differences only exist in *APOE ε4* allele noncarriers (adjusting for only gender and age) (Dominant: OR = 0.733, 95%CI = 0.607–0.884, *Pc* = 0.006; Additive: OR = 0.820, 95%CI = 0.708–0.950, *Pc* = 0.048). Analysis using the recessive models indicate that rs476679 has no effect on the development of LOAD neither in *APOE ε4* allele noncarriers (*P* = 0.764) nor in *APOE ε4* allele carriers after a Bonferroni adjustment (*Pc* = 0.138).

**Figure 1:**
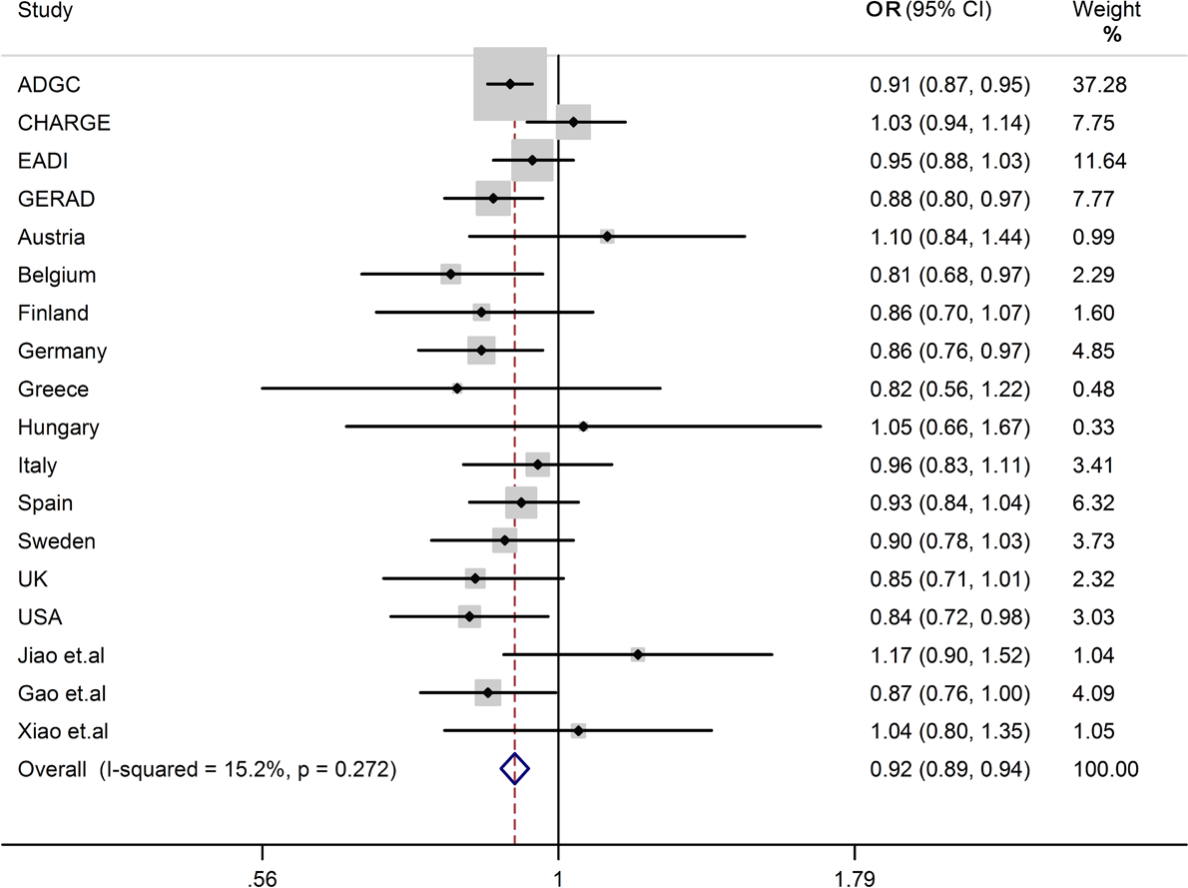
Forest plots for rs1476679 in LOAD and healthy controls in 82525 individuals

We conducted a meta-analysis on the association of rs1476679 and LOAD in a sample of 82525 individuals, and found rs1476679 showed significant association with LOAD (OR = 0.91, 95%CI = 0.89–0.94) (Figure 1) without evident analysis heterogeneity (I^2^ = 20.4%), and the statistical significance of the factors with a low heterogeneity was essentially unchanged from our current study and previous report.

## DISCUSSION

Using a large sample of 922 LOAD patients and 1358 controls, we identified a significant association between the rs1476679 polymorphism and LOAD in a Northern Han Chinese population. Logistic regression analysis reveals that rs1476679 is associated with a decreased risk of LOAD in total sample. We found, after stratifying the subjects by *APOE ε4* status, that this association exists only among *APOE ε4* non-carriers, indicating that rs1476679 is associated with LOAD and can be regarded as an independent protect factor of the AD risk.

The recent large-scale GWAS identified their result (OR = 0.91, 95%CI = 0.89–0.94) that rs1476679 decreased the risk of AD in Caucasian [11]. A following study using a large Spanish sample confirmed the same conclusion through multiple test (OR = 0.846, 95%CI = 0.769–0.932) [16]. However, a set of 1210 samples in Chinese Han and another study of 229 LOAD cases and 318 controls from mainland China found no significant association between rs1476679 and AD [17, 18]. Here, our study identified rs1476679 as a protective factor for LOAD risk. The discrepancy by no means invalidated the initial association and several reasons might attribute to them. The different sample sizes and possibility of false positives could account for this phenomenon. Besides, the complexity of clinical progression, as well as many unknown demographic and clinical variables, such as other medical illnesses and sources of disability, might also cause the bias of results [19]. Furthermore, the effects of some genetic variants confirmed by GWAS, might be population-specific, due to some unknown gene-gene or gene-environment interactions [20]. To avoid these possibly complicated reasons and further investigate these associations, meta-analysis was performed. The results showed that rs1476679 within *ZCWPW1* had a strong association with LOAD.

Regarding to the mechanisms by which the SNP in *ZCWPW1* result to LOAD, rs1476679 may be in LD with functional SNPs in *ZCWPW1* gene. *ZCWPW1* contains the zinc finger CW (zf-CW) domain and the PWWP domain, shows functions in giving rise to chromatin remodeling and methylation states, as well as epigenetic regulations, respectively, indicating *ZCWPW1* as a histone modification reader [12, 21]. As stated by Samantha et al., *ZCWPW1* serves as an eQTL (expression quantitative trait loci) for *GATS, PILRB* and *TRIM4* and affects binding of CTCF and RFX3, a variant in *ZCWPW1* (rs1476679) associated with LOAD risk was considered to have functional relevance [21]. In addition, by activating binding of RFX3, an interesting transcription factor to act on glucokinase gene, we may hypothesis *ZCWPW1* decreases risk of LOAD through suppressing insulin resistance [13].

The region of *ZCWPW1* defined by SNPs associated with LOAD is part of a larger LD block, encompassing many candidate genes [22]. Another possible candidate gene in the *ZCWPW1* region is *NYAP1*, which disruption affects brain size, inhibits neurite elongation and, neuronal morphogenesis [11]. *NYAP1* has role in the regulation of the PI3K signaling pathway in neurons, means it associates with AD risk mainly in this pathway [14]. In addition, *ZNF3* lies at the same locus on chromosome 7 as *ZCWPW1*, thereby it can be regarded as a candidate gene that contains the functional signal in this region [15]. *ZNF3* may interact with BAG3 (involved in ubiquitin/proteasomal functions in protein degradation) and binding of BACH1 (acting on the oxidative stress response and regulating the cell cycle), which are involved in tau pathology of AD. *NYAP1* and *ZNF3* might participate in mechanisms of AD through the above pathways, although no studies reported the association between *NYAP1* or *ZNF3* gene and AD risk [23].

In conclusion, our study provided the first evidence that the carriage of C allele on the *ZCWPW1* (rs1476679) was significant associated with LOAD in a North Han Chinese population. In consistent with the role of *ZCWPW1* (rs1476679) in Caucasians, *ZCWPW1* (rs1476679) also acted as a protective factor to the development of AD in Han Chinese. Further functional examinations into this locus and the region surrounding *ZCWPW1* are required to better elucidate the role of them and their interaction with *ZCWPW1* in AD pathogenesis. Besides, studies in more large cohorts and in other ethnic groups are needed to clarify the role of the locus at *ZCWPW1* in LOAD since genetic variations vary among the populations of different ethnic and geographical origin.

## MATERIALS AND METHODS

### Subjects

Our study investigated 2350 subjects comprising 992 sporadic LOAD patients (mean age at onset: 75.17 ± 6.08 years) and 1358 healthy controls subjects (mean age at examination: 75.49 ± 6.48 years) matched for age and gender. All the LOAD patients and control subjects are unrelated northern Han Chinese residents originally from Shandong Province, which is located in the North of China. The AD patients were recruited from the Department of Neurology at Qingdao Municipal Hospital, and several other 3A-level hospitals in Shandong Province. All patients were subjected to neuropsychological examination, structural neuroimaging consisting of brain computed tomography and/or magnetic resonance imaging. A consensus clinical diagnosis of probable AD was carried out by at least two neurologists according to the criteria of the National Institute of Neurological and Communicative Disorders and Stroke/Alzheimer's Disease and Related Disorders Association (NINCDS/ADRDA) [24]. All the patients are considered to be sporadic because none of their first-degree relatives have dementia in their family history. The information of patients, including age at onset and family history, were determined by their guardians. The age-and gender-matched healthy controls were recruited from the Health Examination Center of the Qingdao Municipal Hospital according to the principles described [25] and were confirmed healthy and neurologically normal by medical history, general examination, laboratory examination, and Mini Mental State Examination (score ≥ 28 points) by physicians and neurologists. Demographic details of the sample set are shown in Table 1. Informed consent was obtained from all subjects or their caregivers, and the protocol of our study was approved by the Institutional Ethics Committees [20].

### Genotyping

Genomic DNA was extracted from the peripheral blood leukocytes of AD patients and healthy individuals using the Wizard genomic DNA purification kit (Cat. #A1125, Promega, USA). Genotyping of *ZCWPW1* (rs1476679) and *APOE* (rs429358 and rs7412) polymorphisms were accomplished by the improved multiplex ligase detection reaction (iMLDR) method, with technical support from the Shanghai Genesky Biotechnology Company (genotyping details are available from the corresponding author) [2]. Data analysis was achieved using GeneMapper Software v4.1 (Applied Biosystems). These DNA samples, which were selected randomly from patients and controls, were sequenced to validate the genotyping using the ligation detection reaction method. Genotyping details are available from the authors upon request.

### Statistical analysis

Statistical analysis was calculated using SPSS16.0 software. Genotype and allele frequencies were computed by counting. Hardy–Weinberg equilibrium was tested using the chi-square test. Disparity of the characteristics between AD patients and control subjects were tested by the Student-*t* test or the χ2 test. The distributions of genotypes and alleles between the two groups were compared using the χ2 test. Differences between the two groups after stratification for *APOE ε4* status were also examined by the χ2 test. For further analysis of the frequencies of the AD patients and control subjects, we defined various genetic models as 1 (CC + CT) versus 0 (TT) for dominant, 1 (CC) versus 0 (CT + TT) for recessive, and 2 (CC) versus 1 (CT) versus 0 (TT) for additive. We then tested them using logistic regression adjusted for age, gender, and *APOE ε4* status (presence or absence of ε4 allele). The significance of an SNP ×*APOE ε4* interaction was also tested for this SNP by logistic regression. The *P* value, odds ratios (ORs) and 95% confidence intervals (CIs) were computed. The criterion for significant difference is *P* < 0.05. A Bonferroni-corrected *P* (*Pc*) value was computed to control false discovery rate. Estimation of the statistical power was performed with STPLAN 4.3 software.

Furthermore, we combined our data with the results from meta-analysis of 74,046 individuals [11] and other reports about *ZCWPW1* (rs1476679) and LOAD [16–18] by fixed-effects inverse variance-weighted methods. Meanwhile, we generated I^2^ estimates with evaluate the possible effect of study heterogeneity on the results. We used Stata V.12.0 to perform all the meta-analyses.
